# The Effect of Postpartum Digital Lactation Counseling on Breastfeeding Outcomes: A Systematic Review

**DOI:** 10.3390/healthcare14213686

**Published:** 2026-10-31

**Authors:** Eda Kaya, Şirvan Özçelik, Gözde Demir Şenbolat, İlkay Güngör Satılmış

**Affiliations:** 1 Department of Midwifery, Faculty of Health Sciences, Atlas University, Istanbul 34408, Türkiye; 2 Department of Women’s Health and Diseases Nursing, Institute of Graduate Studies, Istanbul University-Cerrahpaşa, Istanbul 34320, Türkiye; sirvannozcelik@hotmail.com (Ş.Ö.); gozde.demir@ogr.iuc.edu.tr (G.D.Ş.); 3 Department of Women’s Health and Diseases Nursing, Florence Nightingale Faculty of Nursing, Istanbul University-Cerrahpaşa, Istanbul 34320, Türkiye; ilkay.gungorsatilmis@iuc.edu.tr

**Keywords:** postpartum, digital lactation, telelactation, breastfeeding outcomes, exclusive breastfeeding

## Abstract

**Highlights:**

**What are the main findings?**

- Digital breastfeeding support was associated with higher self-efficacy in several trials, but effects differed by instrument, follow-up time, and intervention design.
- Effects on exclusive breastfeeding varied across trials; randomized comparisons and analyses based on actual application use yielded different conclusions.

**What are the implications of the main findings?**

- Interactive, healthcare-professional-guided digital interventions with regular follow-up and personalized support appear more effective than passive, information-only approaches.
- Digital lactation counseling can be integrated into postpartum care as a complementary strategy to provide accessible and continuous breastfeeding support, particularly when face-to-face services are limited.

**Abstract:**

**Background/Objectives**: This systematic review evaluated the effects of digital lactation counseling interventions delivered during the postpartum period on breastfeeding self-efficacy, breastfeeding success, and exclusive breastfeeding. **Methods**: This systematic review adhered to the PRISMA 2020 guidelines and was registered in PROSPERO (CRD420261442948). The databases PubMed, Google Scholar, Web of Science, Scopus, and MEDLINE were systematically searched for studies published from 2015 to 2025. Eligible studies included randomized controlled trials with accessible full texts that evaluated digital lactation counseling interventions and reported quantitative breastfeeding outcomes. Methodological quality was appraised using the Joanna Briggs Institute critical appraisal checklist, and findings were synthesized narratively. **Results**: Of 881 records identified, 602 remained after deduplication and 38 underwent full-text assessment. Reassessment retained 16 trial reports, including one face-to-face educational intervention with telephone outcome follow-up. Several studies reported higher breastfeeding self-efficacy, but differences in instruments, analyses, and follow-up limited comparison. Exclusive breastfeeding effects varied: some studies favored intervention, whereas other confidence intervals included no difference. Evidence on breastfeeding performance came from two studies using different instruments and did not show a consistent effect. **Conclusions**: Digital lactation support may improve confidence and selected breastfeeding outcomes as an adjunct to postpartum care. The magnitude and durability of benefit remain uncertain because of clinical and methodological heterogeneity, incomplete follow-up and limitations in outcome reporting. Direct comparisons and better standardized outcome assessment are needed before recommending one digital support model over another.

## 1. Introduction

Breastfeeding supports maternal and infant health, with benefits that extend beyond infancy [1]. The World Health Organization (WHO) recommends exclusive breastfeeding for the first 6 months, followed by appropriate complementary feeding with continued breastfeeding [2]. Establishing and maintaining breastfeeding nevertheless requires practical skills, confidence, and timely support. Pain, difficulty with positioning and attachment, and concerns about milk supply can make the early postpartum period particularly challenging. Counseling interventions can help women initiate and continue breastfeeding, although their effects depend on the support provided and the context in which it is delivered [3,4]. Globally, breastfeeding practices remain below recommended levels, and improving them requires sustained investment in supportive services and policies [5]. Postnatal care guidance likewise emphasizes continued support for breastfeeding and maternal well-being after birth [6].

The transition from hospital to home is a critical point in this process. Mothers must apply feeding advice while recovering from childbirth and responding to their infants’ changing needs. Breastfeeding self-efficacy reflects confidence in managing these tasks; it is conceptually distinct from observed feeding performance and from exclusive breastfeeding. Accordingly, improvements in confidence should not be treated as direct evidence of improved milk transfer or sustained exclusive breastfeeding. Digital lactation counseling encompasses breastfeeding education, individualized advice, monitoring, and follow-up delivered through telephone calls, mobile applications, messaging platforms, online groups, or web-based consultations. These formats can extend contact beyond scheduled visits, but access to a digital platform does not necessarily imply access to a clinician or continuous support [7,8]. Internet-based interventions vary in whether they provide information alone or enable interaction with professionals or peers [9]. A previous meta-analysis also examined virtual lactation support and highlighted the importance of specifying the intervention and the breastfeeding outcome under consideration [10]. Syntheses of e-technology- and mobile health-based interventions have reported benefits for selected breastfeeding outcomes [11,12]; however, effects on self-efficacy varied considerably across trials (I^2^ = 88%) [12]. International guidance also frames digital health interventions as a complement to, rather than a substitute for, functioning health systems [13].

A plausible pathway is that demonstrations and repeated access to information improve knowledge, while feedback and problem-solving increase confidence and help mothers address feeding difficulties. These pathways are hypotheses about how support may work; they do not establish which component causes an observed benefit. Digital access, engagement, language, and the availability of appropriate clinical follow-up may influence whether an intervention is usable and effective. The distinction between being offered an application and actually using it matters, especially when interpreting randomized trials [14]. Engagement is not simply a matter of access: it has been conceptualized as both the extent of use and the user’s subjective experience [15], and it has been argued that the goal should be effective engagement, meaning sufficient engagement to achieve the intended outcomes [16]. Digital health may also reduce or reinforce social health inequalities, depending on how accessibility and users’ characteristics are addressed [17].

The expanding range of digital interventions makes it difficult to make a single overall statement about effectiveness. Smartphone interventions can be compared with other digital resources [18], while messaging support can be added to routine hospital education [19]. Other programs combine in-person instruction with postpartum online counseling [20] or begin during pregnancy and continue after birth [21]. These comparisons test different additions to care. Previous reviews establish a relevant evidence base [9,10], but outcome-specific interpretation still requires careful separation of intervention timing, delivery mode, comparator intensity, and measurement instrument. Telelactation, in which mothers consult lactation specialists remotely by video or audio, illustrates this variability: a rural trial showed that it was feasible to implement but found no statistically significant differences in breastfeeding at 12 weeks [22], whereas a larger trial reported modestly higher breastfeeding at 24 weeks, with larger differences among Black participants [23].

This systematic review synthesizes current evidence from randomized controlled trials regarding the effects of digital lactation counseling interventions delivered during the postpartum period on breastfeeding self-efficacy, breastfeeding success, and exclusive breastfeeding. The results are intended to guide the integration of digital breastfeeding support programs into clinical practice and support the development of evidence-based postpartum care services. Given that this review focuses on randomized controlled trials, the underlying research question was structured according to the intervention-focused PICOS framework (Population, Intervention, Comparison, Outcome, Study design): postpartum women (P) receiving digital lactation counseling interventions such as mobile applications, telelactation, video consultations, or chatbot-based counseling (I), compared with standard care, face-to-face counseling, or no additional breastfeeding support (C), with breastfeeding self-efficacy, breastfeeding success, and exclusive breastfeeding as the outcomes of interest (O), assessed exclusively in randomized controlled trials (S). Accordingly, this systematic review sought to answer the following research questions: (i) how do digital lactation counseling interventions delivered during the postpartum period affect breastfeeding outcomes compared with women who do not receive such support, and (ii) do breastfeeding self-efficacy, breastfeeding success scores, and exclusive breastfeeding rates differ between postpartum women receiving digital lactation counseling and those receiving standard care? Building on this rationale, the present systematic review aimed to evaluate the effects of digital lactation counseling interventions delivered during the postpartum period on breastfeeding outcomes.

## 2. Materials and Methods

### 2.1. Aim of the Study

This systematic review evaluated the effects of digital lactation counseling interventions delivered during the postpartum period on breastfeeding outcomes.

The review was conducted and reported in accordance with the Preferred Reporting Items for Systematic Reviews and Meta-Analyses (PRISMA) 2020 statement. The completed PRISMA 2020 checklist is provided in the Supplementary Materials (Table S1). The review protocol was prospectively registered with PROSPERO on 6 July 2026 (CRD420261442948) before data extraction and is publicly accessible through the PROSPERO registry. The final database search was completed in August 2026. No amendments were made to the registered protocol.

### 2.2. Review Questions

The introduction presents the review questions and the intervention-focused PICOS framework underlying this systematic review, and Table 1 summarizes them for clarity and reproducibility.

### 2.3. Search Strategy

PubMed, Google Scholar, Web of Science, Scopus, and MEDLINE were searched using the reported core expression: (“breastfeeding” OR “lactation”) AND (“telelactation” OR “digital health” OR “mobile health” OR “mHealth” OR “mobile application” OR “online counseling” OR “web-based”) AND (“postpartum” OR “post-natal”). The publication window was 1 January 2015 to 31 December 2025, with English and Turkish language full-text reports considered eligible. Google Scholar was treated as a supplementary search source.

### 2.4. Eligibility Criteria

Eligibility criteria were defined using the Population, Intervention, Comparator, Outcomes, and Study Design (PICOS) framework. Studies were eligible if they included women in the postpartum period and evaluated a digitally delivered intervention to provide lactation counseling, breastfeeding education, or breastfeeding support. Eligible digital interventions included, but were not limited to, mobile health applications, web-based programs, telelactation or telehealth services, video-based education, instant messaging platforms, and other digitally mediated breastfeeding support interventions.

Studies were required to report at least one quantitative breastfeeding-related outcome, including breastfeeding self-efficacy, breastfeeding success or effectiveness, or exclusive breastfeeding. Randomized controlled trials comparing a digital intervention with usual care, standard care, face-to-face support, another intervention, or a control condition were eligible. Studies including interventions initiated during pregnancy were considered eligible only when the intervention continued into the postpartum period and breastfeeding-related postpartum outcomes were reported.

Only full-text, peer-reviewed articles published in English or Turkish between 2015 and 2025 were included. Studies were excluded if they (i) used non-randomized, quasi-experimental, observational, or qualitative designs; (ii) evaluated exclusively face-to-face or other non-digital interventions; (iii) were restricted to the antenatal period without a postpartum intervention or postpartum breastfeeding outcome; (iv) did not report quantitative breastfeeding-related outcomes; or (v) focused solely on technology acceptance, usability, user satisfaction, or application development without evaluating breastfeeding outcomes. Conference abstracts, editorials, commentaries, letters, book chapters, theses/dissertations, and publications for which the full text was unavailable were also excluded. Table 2 summarizes the predefined eligibility criteria.

### 2.5. Study Selection

All records identified through database searches were imported into Zotero reference management software (version 7.0), where duplicates were identified and removed. Two researchers independently screened the titles and abstracts of all retrieved records. The same researchers then independently assessed the full texts of potentially eligible studies. The researchers resolved disagreements through discussion and consensus.

**Figure 1:**
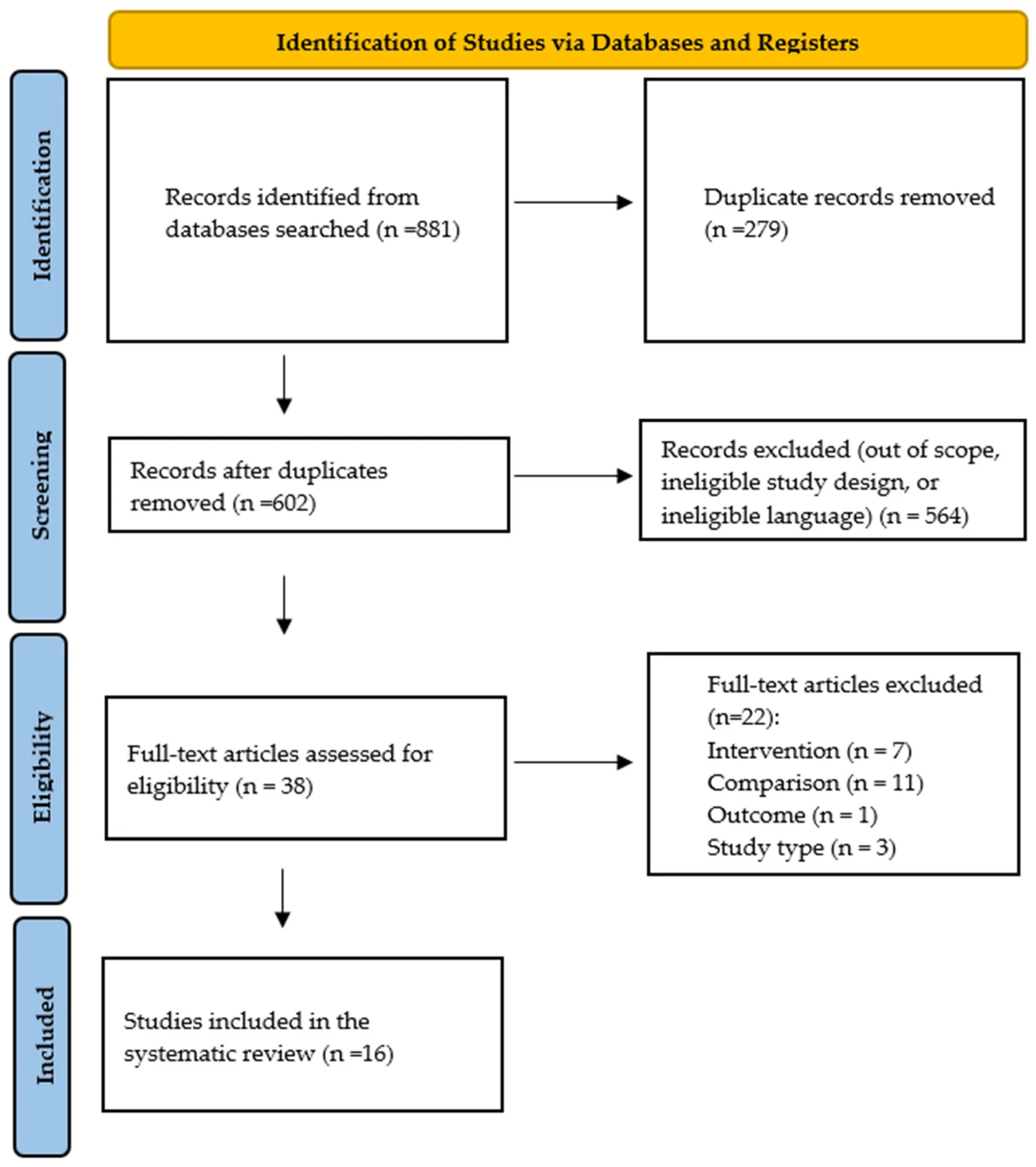
PRISMA Flow Diagram [24].

Database searches identified 881 records (PubMed, *n* = 228; Google Scholar, *n* = 223; Web of Science, *n* = 194; Scopus, *n* = 89; and MEDLINE, *n* = 147). After removing 279 duplicate records, 602 records underwent title and abstract screening. At this stage, 564 records were excluded because they were outside the review scope, did not meet the predefined population or study design criteria, or were published in an ineligible language. The remaining 38 full-text articles were assessed in detail for eligibility; 22 were excluded for not meeting the eligibility criteria. Ultimately, 16 studies met the predefined criteria and were included in the systematic review. The PRISMA flow diagram (Figure 1) presents the study selection and screening process in detail [24].

### 2.6. Data Extraction and Synthesis

Data were extracted into a structured study-level record covering design, setting, population, intervention, comparator, outcome definition, instrument, follow-up and analysis. A structured narrative synthesis of quantitative results, without meta-analysis, grouped findings by self-efficacy, breastfeeding performance and exclusive breastfeeding. Within each domain, follow-up, comparator, measurement instrument and analysis population were considered before comparing effects. Effect magnitude and uncertainty were prioritized over counting statistically significant results. The reporting of study grouping, effect metrics, and synthesis limitations followed the Synthesis Without Meta-analysis (SWiM) guideline [25].

### 2.7. Quality Assessment

This systematic review assessed the methodological quality of the included studies using the critical appraisal tool developed by the Joanna Briggs Institute (JBI). The JBI Critical Appraisal Checklist for Randomized Controlled Trials includes 13 items, and more positive responses indicate higher methodological quality [26]. During appraisal, independent reviewers rated each item as “Yes,” “No,” “Unclear,” or “Not applicable.” A “Yes” response indicated that the relevant criterion was clearly met and appropriately addressed in the study; “No” indicated that the criterion was not met or was inadequately addressed; “Unclear” indicated insufficient reporting to determine whether the criterion had been met; and “Not applicable” indicated that the criterion was not relevant to the specific study design and was therefore excluded from the assessment.

Reviewers resolved any disagreements during the appraisal process through discussion and consensus. Methodological quality scores for each included study were calculated based on the JBI checklist, and the results are presented in the Supplementary Materials (Table S2).

## 3. Results

The findings were organized using a structured narrative synthesis of quantitative trial results without meta-analysis. Studies were grouped by outcome domain, then compared by instrument, follow-up, and comparator. Reported and reconstructed effects were interpreted separately, with attention to confidence intervals, missing outcomes and methodological limitations. Table 2 presents the study-level comparisons underpinning this synthesis; repeated rows represent different outcomes or assessments from the same trial, rather than independent studies.

### 3.1. Study Characteristics

This systematic review included 16 randomized controlled trials published between 2018 and 2025 that met the eligibility criteria. Table 3 summarizes the sample characteristics, interventions, data collection instruments, follow-up time points, and key findings of the included studies. Five studies were conducted in Türkiye [19,20,21,27,28], three in Brazil [29,30,31], two each in China [14,32] and Iran [33,34], and one each in Spain [35], Malaysia [36], Hong Kong [37] and the United States [8]. Sample sizes ranged from 66 [28] to 251 [29] participants, with approximately 2150 participants across the included studies.

The digital lactation counseling interventions evaluated in the included studies varied considerably in content. The interventions could be categorized as smartphone- or mobile application–based education and follow-up [8,14,28,33,34], support delivered via instant messaging platforms such as WhatsApp [19,32], web-based or video-assisted online education [21,37], telephone-based counseling [31,35], support groups delivered through online social networks [29] and hybrid digital counseling interventions combining face-to-face education with digital support [20,27,30,36]. Breastfeeding outcomes were primarily reported as breastfeeding self-efficacy, most commonly assessed with the Breastfeeding Self-Efficacy Scale–Short Form (BSES-SF) [38]; breastfeeding success, evaluated with the LATCH and IMDAT assessment tools; and the rate and/or duration of exclusive breastfeeding.

### 3.2. Breastfeeding Self-Efficacy

Several trials reported higher self-efficacy scores in intervention groups, but the difference varied substantially. In Velioğlu and Demirci, the reconstructed MD was 26.93 points at three months (95% CI 25.19–28.67) and 31.88 at six months (95% CI 31.33–32.43) [21]. Karaahmet and Bilgiç reported six-month means of 67.56 and 52.53, corresponding to an unadjusted MD of 15.03 (95% CI 13.10–16.96) [20]. Wong and Chien showed smaller endpoint differences: MD 6.91 (95% CI 2.23–11.59) at two months and 7.20 (95% CI 1.50–12.90) at six months [37].

In the three-arm trial by Karaçay Yıkar and Nazik, the seven-week e-mobile mean was 62.16, compared with 46.08 under routine care; the reconstructed MD was 16.08 (95% CI 12.00–20.16). The reported omnibus test covered all three groups, and the home-care group had the highest mean; therefore, this study does not establish digital care as superior to home care [27]. Seddighi et al. reported endpoint means of 53.78 and 41.90 after the eight-week intervention, yielding a reconstructed MD of 11.88 (95% CI 7.41–16.35). Their source table provides within-group change tests, so those *p*-values were not assigned to the between-group endpoint difference [34]. Sarı Öztürk and Demir reported little difference at one week (52.06 versus 51.57; *p* = 0.58) and a larger difference at two months (64.21 versus 50.24), but ambiguous dispersion prevented reliable CI reconstruction [28].

Other findings depended on the analysis selected. Jia et al. reported changes of 0.13 and 0.33 in the originally assigned intervention and control groups, respectively (*p* = 0.94), providing no clear evidence of improved self-efficacy [14]. In Mohamad Pilus et al., eight-week means were 53.34 and 51.89 (*p* = 0.37) [36].

Seyyedi et al. reported higher endpoint scores after a three-month intervention (145.92 versus 125.50; *p* < 0.001), but the instrument was described as BSES-SF despite totals inconsistent with its usual scoring range. These results were retained as reported and were not combined with verified short-form totals [33]. Dodou et al. reported day-180 medians of 67 and 61 (*p* < 0.001 for the reported between-group comparison); substantial attrition and exclusions related to breastfeeding cessation limit interpretation [31]. Araque García et al. reported greater confidence in sufficient milk for one item (84% versus 44%; *p* < 0.001), but inconsistent item denominators and the absence of a verified total-score contrast prevent interpreting this as an overall self-efficacy effect [35].

Javorski et al. reported eighth-week BSES-SF means of 65.8 ± 5.5 and 51.2 ± 17.2, with 56 participants per group (reported *p* < 0.001). The descriptive mean difference was 14.60 points. Because the intervention was a face-to-face flipchart session and telephone contacts collected outcomes, this result supports educational support rather than the effectiveness of digital lactation counseling [30].

### 3.3. Breastfeeding Performance and Milk-Intake Indicators

Two studies measured distinct aspects of breastfeeding performance. Velioğlu and Demirci reported six-month IMDAT means of 9.52 and 6.97, corresponding to a reconstructed MD of 2.55 (95% CI 2.20–2.90; source *p* < 0.001) [21]. Araque García et al. reported no statistically clear differences in the five LATCH component comparisons at day 15; all reported *p*-values exceeded 0.05 [35]. IMDAT reflects indicators of milk in-take, whereas LATCH assesses features of feeding performance. A higher IMDAT score in one trial and uncertain LATCH component differences in another do not establish a common effect on an interchangeable outcome called breastfeeding success.

### 3.4. Exclusive Breastfeeding Rates and Duration

The exclusive breastfeeding comparisons showed benefits in some settings and substantial uncertainty in others. At six months, Korkut Öksüz and İnal reported 40/64 exclusively breastfeeding participants in the intervention group versus 7/65 in the control group, giving a reconstructed RR of 5.80 (95% CI 2.81–11.98) [19]. Karaahmet and Bilgiç reported 40/76 versus 19/75, giving a reconstructed RR of 2.08 (95% CI 1.33–3.24), although the source percentages conflict with these counts [20]. At two months, Sarı Öztürk and Demir reported 29/33 versus 14/33, giving a reconstructed RR of 2.07 (95% CI 1.36–3.14) [28].

Huang et al. reported six-month counts of 17/39 versus 9/40. The reconstructed binary RR was 1.94 (95% CI 0.98–3.81), whereas the source *p* = 0.02 referred to an omnibus comparison of feeding modes. This *p*-value cannot be used to declare the reconstructed binary comparison significant [32]. Wong and Chien reported an adjusted RR of 1.79 at two months (95% CI 1.31–2.45) and 1.78 at six months (95% CI 0.96–3.32; *p* = 0.068) [37]. Similar point estimates with different precision do not, by themselves, show that the intervention effect diminished over time.

Duration analyses provided additional information. Cavalcanti et al. reported a lower hazard of early exclusive breastfeeding interruption (HR 0.38, 95% CI 0.28–0.51; *p* < 0.0001) and median durations of 149 versus 86 days [29]. Its reported sixth-month proportions were 33.3% and 8.3%, but a binary RR was not reconstructed because the corresponding denominators were not explicitly verified. Korkut Öksüz and İnal reported mean durations of 4.75 versus 2.21 months [19], Huang et al. reported medians of 108 versus 89 days [32], and Wong and Chien reported survival-based means of 111.25 versus 71.92 days [37]. These summaries measure related but distinct aspects of continuation and were not combined.

By contrast, Jia et al. found no clear difference in exclusive breastfeeding at two months (OR 1.20, 95% CI 0.57–2.55; *p* = 0.63). The adjusted full-breastfeeding OR of 3.16 (95% CI 1.05–9.54) concerned a broader outcome that includes predominant breastfeeding and should not be interpreted as an exclusive breastfeeding effect [14]. Lewkowitz et al. reported no clear six-month difference when comparing breastfeeding applications (RR 0.77, 95% CI 0.25–2.38; *p* = 0.60) [8]. Thus, the synthesis supports possible benefits from particular support packages while retaining uncertainty about the effect of adding a specific digital component to existing care.

Javorski et al. reported exclusive breastfeeding in 44/56 intervention participants and 20/56 controls at eight weeks (reported RR 2.20, 95% CI 1.51–3.21; *p* < 0.001). This favorable result concerns the antenatal flipchart intervention [30].

## 4. Discussion

### 4.1. Interpretation in the Context of Existing Evidence

The central finding is that digital lactation support may improve confidence and selected breastfeeding outcomes, but it does not produce a uniform effect across programs or outcome domains. Improvements in self-efficacy were more apparent in several individual trials than improvements in measured feeding performance, while estimates of exclusive breastfeeding ranged from substantial benefit to considerable uncertainty. Confidence is relevant to a mother’s ability to manage feeding difficulties, but it is not a direct measure of milk transfer or sustained exclusivity. Araque García et al., for example, reported a favorable self-efficacy item without a clear difference in LATCH components [35]. The present evidence therefore supports interpreting confidence, feeding performance and feeding behavior separately, rather than treating them as successive, demonstrated steps in a single causal pathway. Whether interventions that raise self-efficacy also produce higher breastfeeding rates has been examined in earlier syntheses [39]; the present review adds that, within digitally delivered programs, these outcomes still need to be interpreted separately.

These findings align with, but are more qualified than, earlier reviews. Blackmore et al. reported higher exclusive breastfeeding at one and six months with virtual lactation support [10], while Almohanna et al. identified interaction and personalized content as potentially useful features of internet-based interventions [9]. However, those findings do not identify the contribution of each component in the trials reviewed here. The broader Cochrane review by Gavine et al. found that organized breastfeeding support probably reduces cessation but did not identify clear differential effects by provider or delivery mode; power for such comparisons was limited [40]. Taken together, the evidence favors making effective support accessible, while leaving open whether a digital, face-to-face or combined service is best for a particular population. The pooled evidence on mobile health interventions likewise reported higher exclusive breastfeeding and self-efficacy alongside marked heterogeneity [12], which is consistent with the variation observed here across programs and outcomes. Telelactation trials show a similar pattern: video-based lactation consultations were feasible to implement, but breastfeeding differences were not statistically significant in the earlier rural trial [22] and were modest overall, though larger among Black participants, in the later trial [23].

### 4.2. Clinical and Methodological Heterogeneity

Intervention content and comparator intensity are major sources of heterogeneity. Such comparisons estimate the effect of an entire package, not the independent effect of the technology. Huang’s program also involved partners, while Cavalcanti used an online participatory group, making their treatment contrasts different even though both used digital communication [29,32]. Likewise, a trial adding an application to an existing digital support environment tests a smaller increment of care than one adding intensive counseling to routine services [6,9]. A null incremental result should not be generalized to the absence of benefit from breastfeeding support as a whole. Apparent differences between platforms may instead reflect contact time, professional availability, usual care or co-interventions. The retained Javorski trial adds a further difference: it evaluates face-to-face education with remote outcome collection, so it cannot identify the benefit of delivering support digitally [30].

Delivery and population differences further limit transferability. Live counseling allows immediate questions, whereas asynchronous messages may be easier to access but depend on response time and engagement; these are plausible service mechanisms rather than component effects established in the present trials. Several studies recruited primiparous women, and others focused on women after cesarean delivery or low-income first-time mothers [8,19,20,21,27,28,35,37]. Baseline experience, postoperative difficulties, available family support and access to routine services may alter both the need for support and the opportunity to benefit. The results cannot automatically be extended to multiparous women, medically complex mother–infant pairs or settings with substantially different care pathways. Eligibility requirements for a smartphone, internet access or literacy can also select participants already able to use a digital service, limiting conclusions about those facing the greatest access barriers [14,19]. Accessibility is therefore central to implementation: digital health may reduce or reinforce social health inequalities depending on how access, digital literacy, and users’ needs are addressed [17], and digital interventions are intended to complement, not replace, functioning health services [13].

Outcome definitions and instruments change what the estimates mean. A total self-efficacy score, a favorable response to a single item, and a LATCH component are not equivalent measures; none can substitute for exclusive breastfeeding. The distinction between current feeding status and uninterrupted exclusivity since birth is especially important. WHO/UNICEF measurement guidance distinguishes standardized infant-feeding indicators, including current-status measures based on recent recall [41]. A favorable current-status result does not necessarily mean exclusivity was maintained continuously. Full breastfeeding, as defined in Jia et al., additionally includes predominant breastfeeding [14].

Follow-up schedules and analysis populations also influence comparability. An effect measured eight weeks after an intervention need not reflect the same infant age as an effect measured eight weeks postpartum, and early confidence may change as feeding difficulties, family routines, and employment demands evolve. Longer follow-up can involve more missing outcomes and fewer breastfeeding events. The six-month CI in Wong and Chien was wider than the two-month CI despite very similar adjusted RR estimates, so a change in statistical significance is insufficient evidence that benefit disappeared [37]. In Dodou et al., differential losses and exclusions after breastfeeding cessation may favor the more persistent breastfeeding participants [31]. Available-case results therefore require careful interpretation alongside randomized denominators and reasons for loss; multiple follow-up rows from one trial must not be counted as independent confirmations of benefit. Engagement data add a further layer of uncertainty: trials that report only assignment to a digital program say little about how much of it participants used, and the level of engagement needed to achieve an intended outcome may differ from maximal use [15,16].

### 4.3. Limitations

This review has several limitations related to the included evidence. The included studies varied considerably in terms of intervention content and duration, comparator conditions, outcome measurement tools, and assessment time points, precluding meta-analysis and limiting direct comparability across studies. Sample sizes were generally small to moderate, ranging from 66 to 251 participants. In addition, several studies showed methodological limitations based on the JBI critical appraisal checklist, particularly regarding blinding, allocation concealment, and completeness of outcome reporting. Most studies had relatively short follow-up periods, limiting conclusions regarding the long-term sustainability of intervention effects. The geographical distribution of the included evidence was also uneven, with several regions, including South Asia, Gulf countries, Japan, Australia, several African regions, and parts of Europe, absent or underrepresented among the included RCTs. This limited geographical representation may restrict the global generalizability and transferability of the findings, particularly given differences across settings in breastfeeding practices, healthcare systems, postpartum care pathways, digital access, and the organization of lactation support. Further RCTs from geographically diverse and underrepresented settings are therefore needed to strengthen the global evidence base.

Several limitations also relate to the review process itself. The search was restricted to studies published in English or Turkish between 2015 and 2025, potentially excluding relevant evidence published in other languages or outside this period. Potential bias arising from missing results, including publication bias, was not formally assessed. In addition, the authors did not evaluate the certainty of the evidence using a structured framework such as GRADE. These limitations should be considered when interpreting the findings, and they should be addressed in future updates of this review.

## 5. Conclusions

This systematic review evaluated the effects of digital lactation counseling interventions delivered during the postpartum period on breastfeeding outcomes. The findings suggest that digital lactation counseling may benefit breastfeeding outcomes, particularly breastfeeding self-efficacy. However, its effects on breastfeeding success, breastfeeding duration, and exclusive breastfeeding rates appear to vary according to the type and content of the digital intervention, continuity of support, and the quality of healthcare professional involvement. Structured, interactive digital counseling models with regular follow-up appear more effective at improving breastfeeding outcomes than passive interventions based solely on information delivery. In particular, telelactation, telephone-based counseling, and nurse- or midwife-guided digital support programs appear to support breastfeeding continuation, whereas standalone mobile applications may have more limited effects on long-term breastfeeding outcomes.

Accordingly, digital lactation counseling may be integrated into postpartum care services and delivered under the guidance of nurses and midwives, as healthcare professional involvement may enhance the reliability and sustainability of these services. Accordingly, digital lactation counseling may be considered for integration into postpartum care services and delivered under the guidance of nurses and midwives, as healthcare professional involvement may enhance the reliability and sustainability of such services. Particularly in rural or disadvantaged settings where access to face-to-face counseling is limited, digital interventions may serve as an important complement to ensure continuity of breastfeeding support. In clinical practice, hybrid models that combine regular follow-up with personalized feedback should be prioritized over standalone mobile applications designed mainly for information delivery, as these approaches may support more sustainable breastfeeding outcomes. Implementation planning should also consider the characteristics of the intervention, the setting, the individuals involved, and the implementation process, for example, by using established frameworks such as the Consolidated Framework for Implementation Research [42], and should be aligned with postnatal care recommendations that emphasize continued breastfeeding support [6].

Future research should use standardized outcome measures and follow-up time points, compare the effectiveness of different components of digital interventions, and include studies with larger sample sizes and longer follow-up periods. Such research will help develop evidence-based, accessible, and sustainable digital breastfeeding support models and support their integration into postpartum care services. Trials should also report how much of the digital component participants actually used and examine implementation factors across intervention, setting, individual, and process domains [16,42].

## Figures and Tables

**Table 1:** PICOS Framework and Operational Criteria.

Component	Description
Population	Women with a relevant postpartum breastfeeding outcome.
Intervention	Electronic breastfeeding education, counseling, or support delivered at least partly postpartum: telephone, application, messaging, social media, or web consultation.
Comparator	Usual care, face-to-face support, no additional support, or alternative digital education.
Outcomes	Breastfeeding self-efficacy, breastfeeding success, and exclusive breast-feeding.
Study design	Randomized controlled trials.
Other limits	Full-text reports in English or Turkish, published during 2015–2025. Pregnancy-only interventions and prenatal-only outcomes were excluded.

**Table 2:** Summary of eligibility criteria.

Criterion	Inclusion Criteria	Exclusion Criteria
Population	Women in the postpartum period	Studies without a postpartum population or postpartum component relevant to breastfeeding
Intervention	Digitally delivered lactation counseling, breastfeeding education, or breastfeeding support (e.g., mobile applications, web-based programs, telelactation/telehealth, video-based education, or messaging platforms)	Exclusively face-to-face or other non-digital breastfeeding counseling/support interventions
Comparator	Usual/standard care, routine care, face-to-face support, another intervention, or control condition	—
Outcomes	At least one quantitative breastfeeding-related outcome, including breastfeeding self-efficacy, breastfeeding success/effectiveness, or exclusive breastfeeding	No quantitative breastfeeding-related outcome; studies focused solely on technology acceptance, usability, satisfaction, or application development
Study design	Randomized controlled trials	Non-randomized, quasi-experimental, observational, or qualitative studies
Publication characteristics	Full-text, peer-reviewed articles published in English or Turkish between 2015 and 2025	Conference abstracts, editorials, commentaries, letters, book chapters, theses/dissertations, non-peer-reviewed publications or articles without accessible full text

**Table 3:** Characteristics of the Included Studies.

Study	Outcome	Intervention	Control	Effect/Change	95% CI	*p*-Value	Follow-Up	Direction
Jia et al. 2025 [14]	Exclusive breastfeeding	29/55; 52.7%	26/54; 48.1%	OR 1.20	0.57 to 2.55	0.63	2 months	No clear difference
Lewkowitz et al. 2021 [8]	Exclusive breastfeeding	4/29; 13.8%	7/39; 17.9%	RR 0.77	0.25 to 2.38	0.60	6 months	No clear difference
Korkut Öksüz and İnal 2023 [19]	Exclusive breastfeeding	40/64; 62.5%	7/65; 10.8%	RR 5.80	2.81 to 11.98	0.001	6 months	Favors intervention
Huang et al. 2024 [32]	Exclusive breastfeeding	17/39; 43.6%	9/40; 22.5%	RR 1.94	0.98 to 3.81	0.02	6 months	No clear difference
Cavalcanti et al. 2019 [29]	Exclusive breastfeeding	38; 33.3%	10; 8.3%	Approx. +25.0 percentage points	NR	<0.001	6 months	Higher reported intervention proportion
Sarı Öztürk and Demir 2023 [28]	Exclusive breastfeeding	29/33; 87.9%	14/33; 42.4%	RR 2.07	1.36 to 3.14	NR	2 months	Favors intervention
Karaahmet and Bilgiç 2022 [20]	Exclusive breastfeeding	40/76; 52.6%	19/75; 25.3%	RR 2.08	1.33 to 3.24	0.000	6 months	Favors intervention
Wong and Chien 2025 [37]	Exclusive breastfeeding	53/75; 70.7%	29/71; 40.8%	aRR 1.79	1.31 to 2.45	<0.001	2 months	Favors intervention
	Exclusive breastfeeding	25/75; 33.3%	12/71; 16.9%	aRR 1.78	0.96 to 3.32	0.068	6 months	No clear difference
Velioğlu and Demirci 2024 [21]	BSES total	58.65 ± 1.75; *n* = 60	31.72 ± 6.64; *n* = 60	MD 26.93	25.19 to 28.67	<0.001	3 months	Favors intervention
	BSES total	57.15 ± 1.40; *n* = 60	25.27 ± 1.67; *n* = 60	MD 31.88	31.33 to 32.43	<0.001	6 months	Favors intervention
Karaçay Yıkar and Nazik 2024 [27]	BSES-SF total	62.16 ± 7.66; *n* = 25	46.08 ± 7.05; *n* = 25	MD 16.08	12.00 to 20.16	0.000	7 weeks	Higher e-mobile mean than control
Seddighi et al. 2022 [34]	BSES-SF total	53.78 ± 12.61; *n* = 96	41.90 ± 17.98; *n* = 91	MD 11.88	7.41 to 16.35	Not a source endpoint contrast	8 weeks after intervention	Favors intervention
Karaahmet and Bilgiç 2022 [20]	BSES-SF total	67.56 ± 6.49; *n* = 76	52.53 ± 5.56; *n* = 75	MD 15.03	13.10 to 16.96	0.001	6 months	Favors intervention
Wong and Chien 2025 [37]	BSES-SF total	46.73 ± 12.88; *n* = 75	39.82 ± 15.74; *n* = 71	MD 6.91	2.23 to 11.59	0.006	2 months	Favors intervention
	BSES-SF total	48.03 ± 16.76; *n* = 75	40.83 ± 18.29; *n* = 71	MD 7.20	1.50 to 12.90	0.018	6 months	Favors intervention
Jia et al. 2025 [14]	BSES-SF change	+0.13	+0.33	Difference in reported changes −0.20	NR	0.94	2 months	No clear difference
Araque García et al. 2025 [35]	BSES-SF item: perceived sufficient milk	84%	44%	Approx. +40 percentage points	NR	<0.001	Day 30	Favors intervention
Sarı Öztürk and Demir 2023 [28]	BSES total	52.06	51.57	MD +0.49	NR	0.58	1 week	No clear difference
	BSES total	64.21	50.24	MD +13.97 (descriptive)	NR	0.00	2 months	Higher intervention mean
Seyyedi et al. 2021 [33]	Self-efficacy; scale version unresolved	145.92; *n* = 40	125.50; *n* = 40	Difference +20.42	Difference CI NR	<0.001	After 3-month intervention	Higher intervention mean
Dodou et al. 2021 [31]	BSES-SF median	67 (65.7–69); *n* = 74	61 (59–65.5); *n* = 49	Difference in reported medians +6	NR	<0.001	Day 180	Higher intervention median; cessation exclusions limit inference
Mohamad Pilus et al. 2022 [36]	Self-efficacy; instrument details uncertain	53.34 ± 9.16	51.89 ± 8.44	MD +1.45	−1.58 to 4.20	0.37	8 weeks	No clear endpoint difference
Velioğlu and Demirci 2024 [21]	IMDAT milk-intake indicator	9.52 ± 0.68; *n* = 60	6.97 ± 1.21; *n* = 60	MD 2.55	2.20 to 2.90	<0.001	6 months	Favors intervention

Abbreviations: CI, confidence interval; RR, risk ratio; OR, odds ratio; aRR, adjusted estimate; MD, mean difference; NR, not reported. Values with ± are reported means ± SD, except where no dispersion is shown.

## Data Availability

No new data were created or analyzed in this study. Data sharing is not applicable to this article.

## Supplementary Materials

The following supporting information can be downloaded at: https://www.mdpi.com/article/10.3390/healthcare14213686/s1, Table S1: Completed PRISMA 2020 checklist, Table S2: JBI Critical Appraisal Checklist for Randomized Controlled Trials.

## Abbreviations

The following abbreviations are used in this manuscript:

BCRS	Brief Coparenting Relationship Scale
BFF	Breastfeeding Friend (smartphone application)
BSES-SF	Breastfeeding Self-Efficacy Scale–Short Form
CES-D	Center for Epidemiologic Studies Depression Scale
EPDS	Edinburgh Postnatal Depression Scale
IFPS II	Infant Feeding Practices Study II
IIFAS	Iowa Infant Feeding Attitude Scale
IMDAT	Scoring System for the Amount of Breast Milk Intake by the Infant
JBI	Joanna Briggs Institute
LATCH	LATCH Breastfeeding Assessment Tool
MAS	Maternal Attachment Scale
PBSES	Prenatal Breastfeeding Self-Efficacy Scale
PICOS	Population, Intervention, Comparison, Outcome, Study design
PRISMA	Preferred Reporting Items for Systematic Reviews and Meta-Analyses
PROSPERO	International Prospective Register of Systematic Reviews
PSOC	Parenting Sense of Competence Scale
RCT	Randomized Controlled Trial
RR	Risk Ratio
STAI	State–Trait Anxiety Inventory
