# Supplementary material for: The Effect of Postpartum Digital Lactation Counseling on Breastfeeding Outcomes: A Systematic Review
Source: Healthcare (Basel). 2026 Oct 31;14(21):3686. doi: 10.3390/healthcare14213686 (PMC13762600; doi:10.3390/healthcare14213686)
Supplement: Supplementary file 1 [file healthcare-14-03686-s001.zip › JBI_RCT_appraisal_Table_S2.pdf]

Table S2. JBI Critical Appraisal Checklist for Randomized Controlled Trials

Y = Yes (green), N = No (red), U = Unclear (amber). Items Q1-Q13 follow the JBI checklist (Tufanaru et al., 2020)

Study	Q1	Q2	Q3	Q4	Q5	Q6	Q7	Q8	Q9	Q10	Q11	Q12	Q13	Yes
Jia et al. (2025) [14]	Y	N	Y	N	N	N	Y	Y	Y	Y	U	N	N	6/13
Lewkowitz et al. (2021) [8]	Y	Y	Y	Y	Y	Y	Y	N	N	Y	U	U	Y	9/13
Araque García et al. (2025) [35]	Y	U	U	N	N	N	Y	N	N	Y	U	N	Y	4/13
Korkut and İnal (2023) [19]	Y	U	Y	N	N	N	Y	U	N	Y	Y	Y	Y	7/13
Huang et al. (2024) [32]	Y	U	Y	U	N	Y	Y	N	N	Y	U	N	Y	6/13
Velioğlu and Demirci (2024) [21]	Y	U	N	U	U	U	Y	Y	Y	Y	Y	U	Y	7/13
Cavalcanti et al. (2019) [29]	Y	Y	U	Y	N	N	Y	Y	Y	Y	U	Y	Y	9/13
Javorski et al. (2018) [30]	Y	U	N	U	U	Y	Y	U	N	Y	U	N	N	4/13
Karaçay Yıkar and Nazik (2024) [27]	Y	U	Y	N	N	N	Y	U	U	N	Y	U	Y	5/13
Sarı Öztürk and Demir (2023) [28]	Y	Y	Y	N	N	N	Y	Y	Y	Y	U	U	Y	8/13
Seyyedi et al. (2021) [33]	Y	Y	Y	N	N	Y	Y	U	N	Y	U	U	Y	7/13
Seddighi et al. (2022) [34]	Y	Y	Y	U	N	U	Y	U	N	Y	Y	N	Y	7/13
Karaahmet and Bilgiç (2022) [20]	Y	Y	Y	N	N	N	Y	Y	N	Y	Y	N	Y	8/13
Dodou et al. (2021) [31]	Y	Y	U	U	N	Y	Y	N	N	Y	U	U	Y	6/13
Pilus et al. (2022) [36]	Y	N	Y	U	N	N	Y	N	U	Y	U	U	N	4/13
Wong and Chien (2025) [37]	Y	Y	Y	N	N	Y	Y	Y	Y	Y	U	Y	Y	10/13

Domain-Level Summary Across the 16 Studies

Item	Domain	Yes	No	Unclear	% Yes
Q1	Randomisation	17	0	0	**100%**
Q2	Allocation concealment	8	2	7	**47%**
Q3	Baseline similarity	12	2	3	**71%**
Q4	Participant blinding	2	9	6	**12%**
Q5	Deliverer blinding	1	14	2	**6%**
Q6	Assessor blinding	7	8	2	**41%**
Q7	Identical treatment	17	0	0	**100%**
Q8	Follow-up complete/analysed	7	5	5	**41%**
Q9	ITT analysis	6	9	2	**35%**
Q10	Outcomes measured alike	16	1	0	**94%**
Q11	Outcome reliability	6	0	11	**35%**
Q12	Statistical analysis	3	6	8	**18%**
Q13	Trial design	14	3	0	**82%**

Item key: Q1. Was true randomization used for assignment of participants to treatment groups? Q2. Was allocation to treatment groups concealed? Q3. Were treatment groups similar at the baseline? Q4. Were participants blind to treatment assignment? Q5. Were those delivering treatment blind to treatment assignment? Q6. Were outcomes assessors blind to treatment assignment? Q7. Were treatment groups treated identically other than the intervention of interest? Q8. Was follow up complete and if not, were differences between groups in terms of their follow up adequately described and analyzed? Q9. Were participants analyzed in the groups to which they were randomized? Q10. Were outcomes measured in the same way for treatment groups? Q11. Were outcomes measured in a reliable way? Q12. Was appropriate statistical analysis used? Q13. Was the trial design appropriate, and any deviations from the standard RCT design accounted for in the conduct and analysis of the trial?

Item-Level Rationale for Each Study

Jia et al. (2025) [14] - 6/13

Item	Judgement	Domain	Basis for judgement
Q1	**Yes**	Randomisation	Sequence generated by a research designer; clinic visit days randomly designated as intervention/control days.
Q2	**No**	Allocation concealment	Clinic staff assigned dyads according to the day and knew the allocation.
Q3	**Yes**	Baseline similarity	Balanced except maternal age in the ITT sample (P=.04).
Q4	**No**	Participant blinding	Not blinded (stated by authors).
Q5	**No**	Deliverer blinding	Health-care workers running both WeChat groups knew allocation.
Q6	**No**	Assessor blinding	All outcomes self-reported by unblinded participants.
Q7	**Yes**	Identical treatment	Both arms received the same WeChat education/consultation; the app was the added component.
Q8	**Yes**	Follow-up complete/analysed	Attrition 17.7% (20% vs 15%); reasons and attrition analysis (Appendix 4) reported.
Q9	**Yes**	ITT analysis	ITT was the primary analysis (per-protocol app-user comparison is secondary and non-randomised).
Q10	**Yes**	Outcomes measured alike	Same surveys and time points in both arms.
Q11	**Unclear**	Outcome reliability	BSES-SF reliability taken from external sources only.
Q12	**No**	Statistical analysis	Day-level allocation analysed at individual level; final n (109) below target (126); random imputation of missing data.
Q13	**No**	Trial design	Switch from individual to day-level (cluster-type) randomisation not accounted for in the analysis.

Lewkowitz et al. (2021) [8]* - 9/13 Yes

Item	Judgement	Domain	Basis for judgement
Q1	**Yes**	Randomisation	Computer-generated sequence, permuted blocks of four (parent RCT).
Q2	**Yes**	Allocation concealment	Phones pre-loaded with the assigned app by computer sequence; researchers and participants blinded.
Q3	**Yes**	Baseline similarity	No significant baseline differences in the analysed subset (Table 1).
Q4	**Yes**	Participant blinding	Participants blinded: both arms received a study app on a phone.
Q5	**Yes**	Deliverer blinding	Care providers/researchers blinded; the RA who reviewed the app was not involved in outcomes.
Q6	**Yes**	Assessor blinding	Outcomes collected by surveys/phone by staff blinded to allocation.
Q7	**Yes**	Identical treatment	Identical phones, apps and usual care; only app content differed.
Q8	**No**	Follow-up complete/analysed	Follow-up fell to 29/41 and 39/46 at 6 months; differential loss not analysed.
Q9	**No**	ITT analysis	ITT stated, but 83 of 170 randomised women were excluded after randomisation (post hoc subgroup by feeding intent).
Q10	**Yes**	Outcomes measured alike	Same surveys and time points.
Q11	**Unclear**	Outcome reliability	Adapted IFPS-II; reliability not reported in the study.
Q12	**Unclear**	Statistical analysis	Underpowered (<80%, acknowledged); many secondary outcomes; post hoc subset; p=0.04 described as non-significant.
Q13	**Yes**	Trial design	Parent trial is a standard parallel RCT; this report is a post hoc secondary analysis of a subset.

Araque García et al. (2025) [35] - 4/13 Yes

Item	Judgement	Domain	Basis for judgement
Q1	**Yes**	Randomisation	Program generating pseudo-random numbers.
Q2	**Unclear**	Allocation concealment	Numbers assigned sequentially as women consented; no concealment described (authors list this as a limitation).
Q3	**Unclear**	Baseline similarity	Table 1 pools both groups (N=73); no between-group baseline comparison.
Q4	**No**	Participant blinding	Stated as not possible.
Q5	**No**	Deliverer blinding	One expert nurse (the principal investigator) ran the platform and knew allocation.
Q6	**No**	Assessor blinding	Self-reported online questionnaires; no blinded assessment.
Q7	**Yes**	Identical treatment	Usual care in both arms; intervention arm additionally had the online consultation.
Q8	**No**	Follow-up complete/analysed	17/90 lost (6 vs 11); reasons given, but no analysis of impact; missing values not imputed.
Q9	**No**	ITT analysis	Complete-case analysis (n=73); no ITT.
Q10	**Yes**	Outcomes measured alike	Same instruments and time points.
Q11	**Unclear**	Outcome reliability	Reliability taken from external sources only.
Q12	**No**	Statistical analysis	Dichotomised ordinal scales; many unadjusted tests without multiplicity control; no effect sizes/CIs; attrition reduced sample below plan.
Q13	**Yes**	Trial design	Standard two-arm parallel, individually randomised design.

Korkut and İnal (2023) [19] - 7/13 Yes

Item	Judgement	Domain	Basis for judgement
Q1	**Yes**	Randomisation	Computer-based randomisation (random.org).
Q2	**Unclear**	Allocation concealment	Who allocated and how allocation was concealed is not described; one researcher delivered and collected data.
Q3	**Yes**	Baseline similarity	No significant baseline differences (Tables 1-2).
Q4	**No**	Participant blinding	Participants and researcher not blinded (stated in limitations).
Q5	**No**	Deliverer blinding	One researcher delivered the WhatsApp support.
Q6	**No**	Assessor blinding	The same researcher collected outcomes by phone; unblinded.
Q7	**Yes**	Identical treatment	Same routine postpartum education; intervention added 6-month support (possible contamination acknowledged).
Q8	**Unclear**	Follow-up complete/analysed	21/150 lost (11 vs 10) with reasons; 11 post-randomisation NICU exclusions; no impact analysis.
Q9	**No**	ITT analysis	Analysed n=129 after post-randomisation exclusions; no ITT.
Q10	**Yes**	Outcomes measured alike	Same phone interview dates and instruments.
Q11	**Yes**	Outcome reliability	BES Cronbach alpha 0.87 in this sample.
Q12	**Yes**	Statistical analysis	t-tests, mixed ANOVA with Bonferroni-Dunn, chi-square; planned n (128) achieved.
Q13	**Yes**	Trial design	Standard parallel design.

Huang et al. (2024) [32] - 6/13

Item	Judgement	Domain	Basis for judgement
Q1	Yes	Randomisation	Sequence generated by a random service.
Q2	Unclear	Allocation concealment	Concealment not described.
Q3	Yes	Baseline similarity	No significant baseline differences (completers, N=79).
Q4	Unclear	Participant blinding	Couples called 'blinded' because they were strangers to each other (contamination control); participants knew they had programme access.
Q5	No	Deliverer blinding	Research team delivered the intervention.
Q6	Yes	Assessor blinding	Evaluators reported blinded to allocation (outcomes still self-reported).
Q7	Yes	Identical treatment	Routine care in both arms; intervention added a multicomponent programme.
Q8	No	Follow-up complete/analysed	17 lost; attrition stated as 21.5% (17/79), inconsistent with 96 recruited; no impact analysis.
Q9	No	ITT analysis	Per-protocol analysis stated explicitly.
Q10	Yes	Outcomes measured alike	Same instruments and time points.
Q11	Unclear	Outcome reliability	Reliability largely from external studies; coparenting scale alpha 0.61; EBF by a single self-report item.
Q12	No	Statistical analysis	Sample size at alpha=.10; per-protocol analysis; many outcomes without correction; apparent table errors.
Q13	Yes	Trial design	Standard parallel design (couples analysed separately by sex).

Velioğlu and Demirci (2024) [21] - 7/13

Item	Judgement	Domain	Basis for judgement
Q1	Yes	Randomisation	Random number list (GraphPad) prepared by an independent statistician.
Q2	Unclear	Allocation concealment	List preassigned by order of admission; concealment mechanism not described.
Q3	No	Baseline similarity	Significant baseline differences in age, gestational week, education, family type, employment, planned pregnancy and antenatal visits (p<.005).
Q4	Unclear	Participant blinding	Blinding not reported; intervention was a website.
Q5	Unclear	Deliverer blinding	Not reported; researcher offered counselling.
Q6	Unclear	Assessor blinding	Not reported; self-report scales.
Q7	Yes	Identical treatment	Control group received no website information; website was the added component.
Q8	Yes	Follow-up complete/analysed	No loss at any follow-up (60 vs 60), as reported.
Q9	Yes	ITT analysis	All 120 analysed as randomised.
Q10	Yes	Outcomes measured alike	Same scales and time points.
Q11	Yes	Outcome reliability	Cronbach alpha computed in this study.
Q12	Unclear	Statistical analysis	Repeated t-tests/RM-ANOVA not adjusted for baseline imbalances; multiple comparisons.
Q13	Yes	Trial design	Standard parallel design.

Cavalcanti et al. (2019) [29] - 9/13 Yes

Item	Judgement	Domain	Basis for judgement
Q1	Yes	Randomisation	Excel random 0/1 list prepared before the study (simple randomisation).
Q2	Yes	Allocation concealment	Recruiters not involved in assignment; nutritionist assigning had no contact with mothers.
Q3	Unclear	Baseline similarity	Similar except number of prenatal consultations (>6: 76% vs 90%).
Q4	Yes	Participant blinding	Mothers unaware that two groups existed or of their allocation (plausibility limited for IG members).
Q5	No	Deliverer blinding	Research team knew allocation (stated).
Q6	No	Assessor blinding	Phone interviews by staff aware of groups.
Q7	Yes	Identical treatment	Same booklet and routine care; intervention added a Facebook group.
Q8	Yes	Follow-up complete/analysed	8 losses (7 vs 1), mostly refusals; censored in the analysis.
Q9	Yes	ITT analysis	All women analysed as randomised (censored at loss).
Q10	Yes	Outcomes measured alike	Same monthly phone interviews.
Q11	Unclear	Outcome reliability	EBF from phone recall using MoH markers; reliability not reported; date imprecision acknowledged.
Q12	Yes	Statistical analysis	Kaplan-Meier, log-rank, proportional-hazards assumption tested; sample size calculated and exceeded.
Q13	Yes	Trial design	Standard parallel design.

Javorski et al. (2018) [30] - 4/13

Item	Judgement	Domain	Basis for judgement
Q1	Yes	Randomisation	Clusters (territories) drawn at random.
Q2	Unclear	Allocation concealment	Two-stage draws unclear; concealment not described.
Q3	No	Baseline similarity	Age differed (<=19 y: 35.7% CG vs 16.1% IG; p=0.002).
Q4	Unclear	Participant blinding	Not reported (individual flipchart counselling).
Q5	Unclear	Deliverer blinding	Not reported.
Q6	Yes	Assessor blinding	Trained research assistant blinded to allocation at telephone follow-up.
Q7	Yes	Identical treatment	Routine prenatal care in both arms; flipchart counselling added.
Q8	Unclear	Follow-up complete/analysed	20/132 lost (10 vs 10); reasons limited; no impact analysis.
Q9	No	ITT analysis	Completers analysed (56 vs 56).
Q10	Yes	Outcomes measured alike	Same instruments and time points.
Q11	Unclear	Outcome reliability	BSES-SF validation cited (alpha 0.7); not computed in this study.
Q12	No	Statistical analysis	Cluster allocation analysed at individual level; no adjustment for age imbalance.
Q13	No	Trial design	Cluster randomisation (4 clusters) not reflected in the analysis.

Karaçay Yıkar and Nazik (2024) [27] - 5/13

Item	Judgement	Domain	Basis for judgement
Q1	Yes	Randomisation	Block randomisation with random combinations; lots drawn.
Q2	Unclear	Allocation concealment	Not described.
Q3	Yes	Baseline similarity	Groups reported homogeneous (P>.05), although education (P=.070) and day-1 BSES (P=.052) were borderline.
Q4	No	Participant blinding	Blinding could not be implemented (stated).
Q5	No	Deliverer blinding	Researcher delivered care/consultancy.
Q6	No	Assessor blinding	Only the statistician was blinded; data collected by the researcher.
Q7	Yes	Identical treatment	Standard care in all groups; home visits or app were the added components.
Q8	Unclear	Follow-up complete/analysed	No attrition reported; flow chart referenced but not presented.
Q9	Unclear	ITT analysis	ITT not addressed.
Q10	No	Outcomes measured alike	Data collected face-to-face, via app or by phone depending on group.
Q11	Yes	Outcome reliability	Cronbach alpha computed in this study (0.82; 0.83).
Q12	Unclear	Statistical analysis	Separate group comparisons at 9 time points; no repeated-measures model despite RM-ANOVA sample-size basis.
Q13	Yes	Trial design	Three-arm parallel design.

Sarı Öztürk and Demir (2023) [28] - 8/13 Yes

Item	Judgement	Domain	Basis for judgement
Q1	Yes	Randomisation	randomizer.org, from a stratified pool.
Q2	Yes	Allocation concealment	Independent nurse randomised; sequence in sealed opaque envelope.
Q3	Yes	Baseline similarity	Table 2: no differences (e.g., age p=0.93).
Q4	No	Participant blinding	Double blinding not possible (stated); blinding only until study start.
Q5	No	Deliverer blinding	Researcher delivered the programme.
Q6	No	Assessor blinding	Only the statistician was blinded; researcher measured infants; Google Forms self-report.
Q7	Yes	Identical treatment	Routine care in both arms; mandala/WhatsApp programme with phone counselling added.
Q8	Yes	Follow-up complete/analysed	No losses after randomisation (66/66; Fig. 1 label 'lost n=33' is erroneous).
Q9	Yes	ITT analysis	All randomised women analysed.
Q10	Yes	Outcomes measured alike	Same instruments and time points.
Q11	Unclear	Outcome reliability	Reliability from literature only.
Q12	Unclear	Statistical analysis	t-test/Mann-Whitney/chi-square; no adjustment; sample-size description ('80% confidence interval') unclear.
Q13	Yes	Trial design	Standard parallel design.

Seyyedi et al. (2021) [33] - 7/13 Yes

Item	Judgement	Domain	Basis for judgement
Q1	**Yes**	Randomisation	Web-based randomisation tool, 1:1.
Q2	**Yes**	Allocation concealment	Randomiser blind to participants and not involved in recruitment.
Q3	**Yes**	Baseline similarity	No significant differences (residency p=0.08; baseline BSE p=0.281).
Q4	**No**	Participant blinding	Participants necessarily aware of the app.
Q5	**No**	Deliverer blinding	Midwives prompting app use knew allocation.
Q6	**Yes**	Assessor blinding	Data collectors reported blinded (questionnaires self-completed).
Q7	**Yes**	Identical treatment	Routine care in both arms; app plus weekly calls added.
Q8	**Unclear**	Follow-up complete/analysed	22/102 lost (11 vs 11); reasons generic; no impact analysis; Fig. 1 arm labels swapped.
Q9	**No**	ITT analysis	ITT stated but only 80 completers analysed.
Q10	**Yes**	Outcomes measured alike	Same questionnaire pack and time points.
Q11	**Unclear**	Outcome reliability	Reliability not reported in this study.
Q12	**Unclear**	Statistical analysis	Underpowered after attrition; BSES means (119-146) exceed the BSES-SF range (14-70), so scale/reporting inconsistent.
Q13	**Yes**	Trial design	Standard parallel design.

Seddighi et al. (2022) [34] - 7/13 Yes

Item	Judgement	Domain	Basis for judgement
Q1	**Yes**	Randomisation	Block randomisation software.
Q2	**Yes**	Allocation concealment	Sealed, locked envelopes kept at centres; blocks drawn daily.
Q3	**Yes**	Baseline similarity	Table 1 similar (P>.05); baseline BSES 48.3 vs 49.1.
Q4	**Unclear**	Participant blinding	Not reported.
Q5	**No**	Deliverer blinding	Researcher taught the app and provided support.
Q6	**Unclear**	Assessor blinding	Not reported.
Q7	**Yes**	Identical treatment	Routine care in both arms; app, SMS reminders and researcher support added.
Q8	**Unclear**	Follow-up complete/analysed	11/198 lost (3 vs 8) with reasons; no analysis.
Q9	**No**	ITT analysis	Completers analysed (96 vs 91).
Q10	**Yes**	Outcomes measured alike	Same scale and time points.
Q11	**Yes**	Outcome reliability	Cronbach alpha 0.94 in this study.
Q12	**No**	Statistical analysis	Between-group comparison not reported (within-group only); sample-size formula garbled; control-group decline unexplained.
Q13	**Yes**	Trial design	Standard parallel design.

Karaahmet and Bilgiç (2022) [20] - 8/13 Yes

Item	Judgement	Domain	Basis for judgement
Q1	Yes	Randomisation	randomizer.org.
Q2	Yes	Allocation concealment	Randomising researcher did not share allocation with the delivering researcher.
Q3	Yes	Baseline similarity	No baseline differences per authors.
Q4	No	Participant blinding	Not blinded.
Q5	No	Deliverer blinding	Counselling delivered by a researcher who knew allocation.
Q6	No	Assessor blinding	Randomisation and data collection by the same researcher (knew allocation).
Q7	Yes	Identical treatment	Routine care in both arms; intervention added demonstration and 6-month online counselling.
Q8	Yes	Follow-up complete/analysed	7/158 lost (3 vs 4); reasons described.
Q9	No	ITT analysis	151/158 analysed (completers).
Q10	Yes	Outcomes measured alike	Same instruments and time points.
Q11	Yes	Outcome reliability	Cronbach alpha 0.92 in this study.
Q12	No	Statistical analysis	Inconsistent reporting (text vs abstract vs tables, p-values, sample-size parameters); inference unreliable.
Q13	Yes	Trial design	Standard parallel design.

Dodou et al. (2021) [31] - 6/13

Item	Judgement	Domain	Basis for judgement
Q1	Yes	Randomisation	Block randomisation (SPSS) by a blinded statistician.
Q2	Yes	Allocation concealment	Group codes held by the statistician until study end.
Q3	Unclear	Baseline similarity	Baseline BSES medians differed (CG 59 vs IG 58; p=0.048).
Q4	Unclear	Participant blinding	'Blinding' claimed by giving the IG an additional intervention; implausible.
Q5	No	Deliverer blinding	Delivered only by the principal investigator.
Q6	Yes	Assessor blinding	Trained assistants blinded to allocation (telephone outcome assessment).
Q7	Yes	Identical treatment	Standard care in both arms; telephone intervention added.
Q8	No	Follow-up complete/analysed	About half lost by 180 days (IG 74 vs CG 49 analysed); reasons limited; no analysis.
Q9	No	ITT analysis	Only respondents analysed; no ITT.
Q10	Yes	Outcomes measured alike	Same scale and telephone mode in both arms.
Q11	Unclear	Outcome reliability	Reliability not reported in this study.
Q12	Unclear	Statistical analysis	Non-parametric tests appropriate, but heavy attrition and baseline imbalance not handled; fewer participants than planned.
Q13	Yes	Trial design	Standard parallel design.

Pilus et al. (2022) [36] - 4/13

Item	Judgement	Domain	Basis for judgement
Q1	Yes	Randomisation	Clinics randomised (Stat Trek) by a nurse not involved in the study.
Q2	No	Allocation concealment	Participants recruited after clinic allocation was known; recruitment bias possible.
Q3	Yes	Baseline similarity	No significant baseline differences.
Q4	Unclear	Participant blinding	Described as blinded, but participants attended the intervention.
Q5	No	Deliverer blinding	Researchers delivered the intervention.
Q6	No	Assessor blinding	Self-completed questionnaires; no assessor blinding.
Q7	Yes	Identical treatment	Usual counselling and pamphlets in control; SeBF added.
Q8	No	Follow-up complete/analysed	Dropout 31% (control) vs 7%; no impact analysis.
Q9	Unclear	ITT analysis	ITT claimed; only 139/172 contributed complete data.
Q10	Yes	Outcomes measured alike	Same instruments and time points.
Q11	Unclear	Outcome reliability	Reliability from external studies.
Q12	Unclear	Statistical analysis	GLMM with covariates; handling of clustering not described; only 12 clusters.
Q13	No	Trial design	Cluster design reflected in sample size (ICC, design effect) but not clearly in the analysis.

Wong and Chien (2025) [37] - 10/13

Item	Judgement	Domain	Basis for judgement
Q1	Yes	Randomisation	Computerised block randomisation, random block size 4/6/8.
Q2	Yes	Allocation concealment	Sealed opaque envelopes by one RA; allocation list by another.
Q3	Yes	Baseline similarity	No baseline differences in socio-demographics or BSE; differed on infant maturity and other talks (adjusted).
Q4	No	Participant blinding	Participants not blinded (stated in limitations).
Q5	No	Deliverer blinding	Midwife/lactation consultant delivered the programme; not blinded.
Q6	Yes	Assessor blinding	Assessor-blinded design; blinded RA analysed data; outcomes self-reported online.
Q7	Yes	Identical treatment	Usual care in both arms; REST added (other-talk attendance adjusted).
Q8	Yes	Follow-up complete/analysed	4/150 lost (2.7%); censored.
Q9	Yes	ITT analysis	ITT principle stated.
Q10	Yes	Outcomes measured alike	Same online surveys and time points.
Q11	Unclear	Outcome reliability	BSES-SF reliability (alpha .95) from prior validation; not clearly recomputed.
Q12	Yes	Statistical analysis	Log-binomial, GEE, survival analysis; confounders adjusted; sample size calculated.
Q13	Yes	Trial design	Standard parallel design.
