# Supplementary material for: The Effect of Postpartum Digital Lactation Counseling on Breastfeeding Outcomes: A Systematic Review
Source: Healthcare (Basel). 2026 Oct 31;14(21):3686. doi: 10.3390/healthcare14213686 (PMC13762600; doi:10.3390/healthcare14213686)
Supplement: Supplementary file 1 [file healthcare-14-03686-s001.zip › Supplementary_Table_S1_PRISMA_06.10.2026.pdf]

Supplementary Table S1. PRISMA 2020 Checklist

Section and Topic	Item #	Checklist item	Location where item is reported
**TITLE**			
Title	1	Identify the report as a systematic review.	Page 1, title.
**ABSTRACT**			
Abstract	2	See the PRISMA 2020 for Abstracts checklist.	Pages 1–2, Abstract.
**INTRODUCTION**			
Rationale	3	Describe the rationale for the review in the context of existing knowledge.	Pages 2–3, Introduction.
Objectives	4	Provide an explicit statement of the objective(s) or question(s) the review addresses.	Page 3, Introduction (objectives, review questions and PICOS); Materials and Methods, Aim of the Study.
**METHODS**			
Eligibility criteria	5	Specify the inclusion and exclusion criteria for the review and how studies were grouped for the syntheses.	Pages 4–5, Eligibility Criteria and Table 2, “Summary of eligibility criteria”; pages 6–7, Data Extraction and Synthesis (outcome grouping).
Information sources	6	Specify all databases, registers, websites, organisations, reference lists and other sources searched or consulted to identify studies. Specify the date when each source was last searched or consulted.	Page 4, Search Strategy (five sources); page 4, Aim of the Study (final search: August 2026). Exact last-search dates for each source are not reported.
Search strategy	7	Present the full search strategies for all databases, registers and websites, including any filters and limits used.	Page 4, Search Strategy (core search expression, publication window and language limits). Full source-specific strategies are not reported.
Selection process	8	Specify the methods used to decide whether a study met the inclusion criteria of the review, including how many reviewers screened each record and each report retrieved, whether they worked independently, and if applicable, details of automation tools used in the process.	Pages 5–6, Study Selection (two independent reviewers at both screening stages; consensus).
Data collection process	9	Specify the methods used to collect data from reports, including how many reviewers collected data from each report, whether they worked independently, any processes for obtaining or confirming data from study investigators, and if applicable, details of automation tools used in the process.	Page 6, Data Extraction and Synthesis (structured extraction record). Number and independence of extractors and investigator-contact procedures are not reported.
Data items	10a	List and define all outcomes for which data were sought. Specify whether all results that were compatible with each outcome domain in each study were sought (e.g. for all measures, time points, analyses), and if not, the methods used to decide which results to collect.	Page 6, Data Extraction and Synthesis; pages 8–11, Table 2, “Characteristics of the Included Studies”; pages 12–13, outcome-specific Results. Rules for selecting among all eligible measures, time points and analyses are not fully reported.
	10b	List and define all other variables for which data were sought (e.g. participant and intervention characteristics, funding sources). Describe any assumptions made about any missing or unclear information.	Page 6, Data Extraction and Synthesis (design, setting, population, intervention, comparator, instrument, follow-up and analysis). Assumptions for missing or unclear information are not explicitly described.
Study risk of bias assessment	11	Specify the methods used to assess risk of bias in the included studies, including details of the tool(s) used, how many reviewers assessed each study and whether they worked independently, and if applicable, details of automation tools used in the process.	Page 7, Quality Assessment (13-item JBI RCT checklist, independent appraisal and consensus); Supplementary Table S2, JBI appraisal. Number of appraisal reviewers is not specified in this section.
Effect measures	12	Specify for each outcome the effect measure(s) (e.g. risk ratio, mean difference) used in the synthesis or presentation of results.	Pages 8–11, Table 2, “Characteristics of the Included Studies” (RR, OR, adjusted estimates, MD and confidence intervals); pages 12–13, outcome-specific Results (also HR and duration measures).
Synthesis methods	13a	Describe the processes used to decide which studies were eligible for each synthesis (e.g. tabulating the study intervention characteristics and comparing against the planned groups for each synthesis (item #5)).	Pages 6–7, Data Extraction and Synthesis; page 7, Results opening paragraph (grouping by outcome, instrument, follow-up and comparator).
	13b	Describe any methods required to prepare the data for presentation or synthesis, such as handling of missing summary statistics, or data conversions.	Page 7, Results opening paragraph (reported and reconstructed effects distinguished); pages 8–13, study table and outcome-specific Results (unverified denominators, ambiguous dispersion and scale inconsistencies).

Section and Topic	Item #	Checklist item	Location where item is reported
			Reconstruction formulas and a complete data-preparation procedure are not reported.
	13c	Describe any methods used to tabulate or visually display results of individual studies and syntheses.	Page 6, Data Extraction and Synthesis (structured study-level records); page 7, Results opening paragraph; pages 8–11, Table 2, "Characteristics of the Included Studies".
	13d	Describe any methods used to synthesize results and provide a rationale for the choice(s). If meta-analysis was performed, describe the model(s), method(s) to identify the presence and extent of statistical heterogeneity, and software package(s) used.	Pages 6–7, Data Extraction and Synthesis (structured narrative synthesis, without meta-analysis; SWiM); page 7, Results opening paragraph; page 15, Limitations (heterogeneity precluded meta-analysis).
	13e	Describe any methods used to explore possible causes of heterogeneity among study results (e.g. subgroup analysis, meta-regression).	Pages 6–7, Data Extraction and Synthesis (qualitative comparison by follow-up, comparator, instrument and analysis population); pages 14–15, Clinical and Methodological Heterogeneity. No formal subgroup analysis or meta-regression is reported.
	13f	Describe any sensitivity analyses conducted to assess robustness of the synthesized results.	Not reported; no formal sensitivity analysis is described.
Reporting bias assessment	14	Describe any methods used to assess risk of bias due to missing results in a synthesis (arising from reporting biases).	Not formally assessed; page 15, Limitations (missing-results and publication bias).
Certainty assessment	15	Describe any methods used to assess certainty (or confidence) in the body of evidence for an outcome.	Not assessed using a structured framework; page 15, Limitations (GRADE not used).
**RESULTS**			
Study selection	16a	Describe the results of the search and selection process, from the number of records identified in the search to the number of studies included in the review, ideally using a flow diagram.	Page 6, Study Selection (881 identified, 279 duplicates, 602 screened, 38 full texts, 22 full-text exclusions and 16 included studies); page 6, Figure 1, PRISMA Flow Diagram.
	16b	Cite studies that might appear to meet the inclusion criteria, but which were excluded, and explain why they were excluded.	Pages 5–6, Study Selection and Figure 1 (aggregate exclusions). Citations and study-specific reasons for apparently eligible excluded studies are not reported.
Study characteristics	17	Cite each included study and present its characteristics.	Page 7, Study Characteristics; pages 8–11, Table 2, "Characteristics of the Included Studies" (study citations, outcomes, group data and time points). Detailed characteristics are described in aggregate in the text.
Risk of bias in studies	18	Present assessments of risk of bias for each included study.	Supplementary Table S2, JBI Critical Appraisal Checklist for Randomized Controlled Trials (study-level matrix and item-level rationales); page 7, Quality Assessment (cross-reference); page 15, Limitations (summary of methodological weaknesses).
Results of individual studies	19	For all outcomes, present, for each study: (a) summary statistics for each group (where appropriate) and (b) an effect estimate and its precision (e.g. confidence/credible interval), ideally using structured tables or plots.	Pages 8–11, Table 2, "Characteristics of the Included Studies"; pages 12–13, outcome-specific Results (group summaries, reported/reconstructed effects and confidence intervals where available; reporting gaps identified).
Results of syntheses	20a	For each synthesis, briefly summarise the characteristics and risk of bias among contributing studies.	Page 7, Study Characteristics; pages 12–13, outcome-specific Results; Supplementary Table S2, study-level JBI assessments; page 15, Limitations. Risk of bias is not summarized separately for each outcome synthesis.
	20b	Present results of all statistical syntheses conducted. If meta-analysis was done, present for each the summary estimate and its precision (e.g. confidence/credible interval) and measures of statistical heterogeneity. If comparing groups, describe the direction of the effect.	Pages 12–13, Breastfeeding Self-Efficacy, Breastfeeding Performance and Milk-Intake Indicators, and Exclusive Breastfeeding Rates and Duration; pages 8–11, study table. Narrative synthesis only; no pooled statistical synthesis was performed.
	20c	Present results of all investigations of possible causes of heterogeneity among study results.	Pages 14–15, Clinical and Methodological Heterogeneity (qualitative interpretation). No formal subgroup analysis or meta-regression results are reported.
	20d	Present results of all sensitivity analyses conducted to assess the robustness of the synthesized results.	Not reported; no formal sensitivity analysis results are presented.
Reporting biases	21	Present assessments of risk of bias due to missing results (arising from reporting biases) for each synthesis assessed.	Not assessed; page 15, Limitations.

Section and Topic	Item #	Checklist item	Location where item is reported
Certainty of evidence	22	Present assessments of certainty (or confidence) in the body of evidence for each outcome assessed.	Not graded; page 15, Limitations.
**DISCUSSION**			
Discussion	23a	Provide a general interpretation of the results in the context of other evidence.	Pages 13–14, Discussion, Interpretation in the Context of Existing Evidence.
	23b	Discuss any limitations of the evidence included in the review.	Pages 14–15, Clinical and Methodological Heterogeneity and Limitations (included evidence).
	23c	Discuss any limitations of the review processes used.	Page 15, Limitations (review process, search restrictions, reporting bias and certainty assessment).
	23d	Discuss implications of the results for practice, policy, and future research.	Pages 15–16, Conclusions (practice and future research implications).
**OTHER INFORMATION**			
Registration and protocol	24a	Provide registration information for the review, including register name and registration number, or state that the review was not registered.	Pages 3–4, Aim of the Study (PROSPERO CRD420261442948; registration on 6 July 2026).
	24b	Indicate where the review protocol can be accessed, or state that a protocol was not prepared.	Pages 3–4, Aim of the Study (protocol publicly accessible through the PROSPERO registry).
	24c	Describe and explain any amendments to information provided at registration or in the protocol.	Page 4, Aim of the Study (no amendments to the registered protocol).
Support	25	Describe sources of financial or non-financial support for the review, and the role of the funders or sponsors in the review.	Page 16, Funding (no external funding). Non-financial support and funder/sponsor roles are not separately described.
Competing interests	26	Declare any competing interests of review authors.	Page 16, Conflicts of Interest.
Availability of data, code and other materials	27	Report which of the following are publicly available and where they can be found: template data collection forms; data extracted from included studies; data used for all analyses; analytic code; any other materials used in the review.	Page 16, Data Availability Statement ("No new data were created or analyzed in this study"); page 16, Supplementary Materials (Tables S1 and S2). Public locations for extraction forms, extracted datasets and analytic code are not provided.

From: Page MJ, McKenzie JE, Bossuyt PM, Boutron I, Hoffmann TC, Mulrow CD, et al. The PRISMA 2020 statement: an updated guideline for reporting systematic reviews. BMJ 2021;372:n71. doi: 10.1136/bmj.n71. This work is licensed under CC BY 4.0. To view a copy of this license, visit https://creativecommons.org/licenses/by/4.0/
